# Isolated oral methotrexate-induced cerebellar leukoencephalopathy: a rare and underrecognized imaging pattern

**DOI:** 10.1055/s-0046-1827043

**Published:** 2026-08-13

**Authors:** Aaliyah Shaikh, Abdulrahman Kareem Sidani, Kevin J. Abrams, Márcio Luís Duarte, Leonardo Furtado Freitas

**Affiliations:** 1 Florida International University, Herbert Wertheim College of Medicine, Miami FL, United States.; 2 University of Florida, Gainesville FL, United States.; 3 Baptist Health South Florida, Radiology Associates of South Florida, Department of Radiology, Miami FL, United States.; 4 Universidade de Ribeirão Preto, Guarujá SP, Brazil.; 5 Diagnósticos da América S.A., São Paulo SP, Brazil.

**Figure 1:**
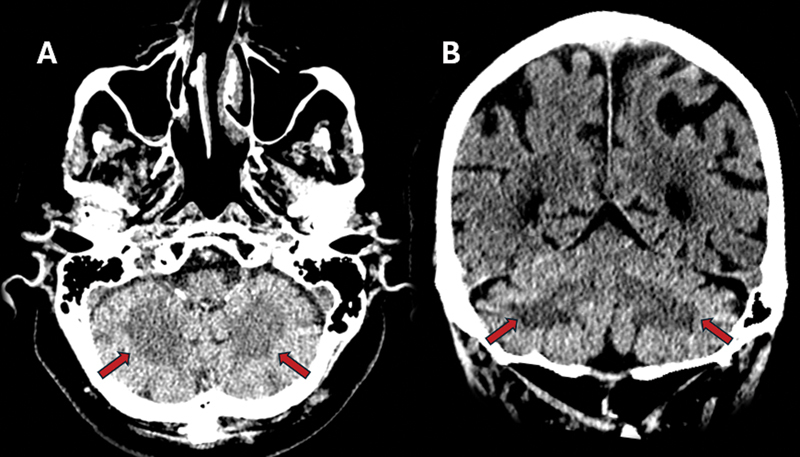
Non-contrast brain computed tomography (CT) scan, axial ( **A** ) and coronal ( **B** ) images. Bilateral and symmetric hypoattenuation in the deep cerebellar white matter surrounding the dentate nuclei (red arrows).

**Figure 2:**
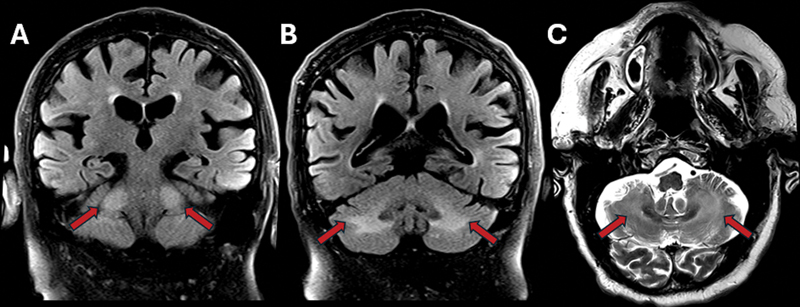
Brain magnetic resonance imaging (MRI) scan, coronal fluid-attenuated inversion recovery (FLAIR) ( **A,B** ) and axial T2-weighted ( **C** ) images. Greater conspicuity of the abnormality in the middle cerebellar peduncles and deep cerebellar white matter surrounding the dentate nuclei, characterized by T2/FLAIR hypersignal consistent with vasogenic edema (red arrows).

**Figure 3:**
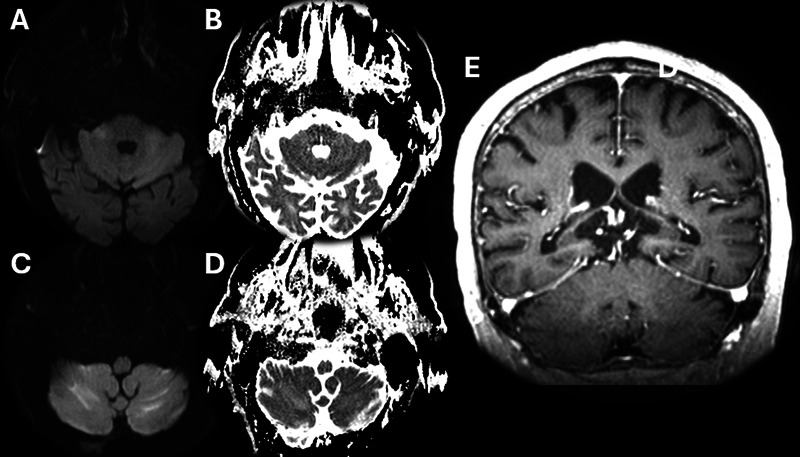
Brain MRI scan, axial diffusion-weighted imaging ( **A,C** ), axial apparent diffusion coefficient (ADC) maps ( **B,D** ), and coronal postcontrast T1 gradient-echo sequence ( **E** ). No restricted diffusion or enhancement in the affected cerebellar regions.

**Figure 4:**
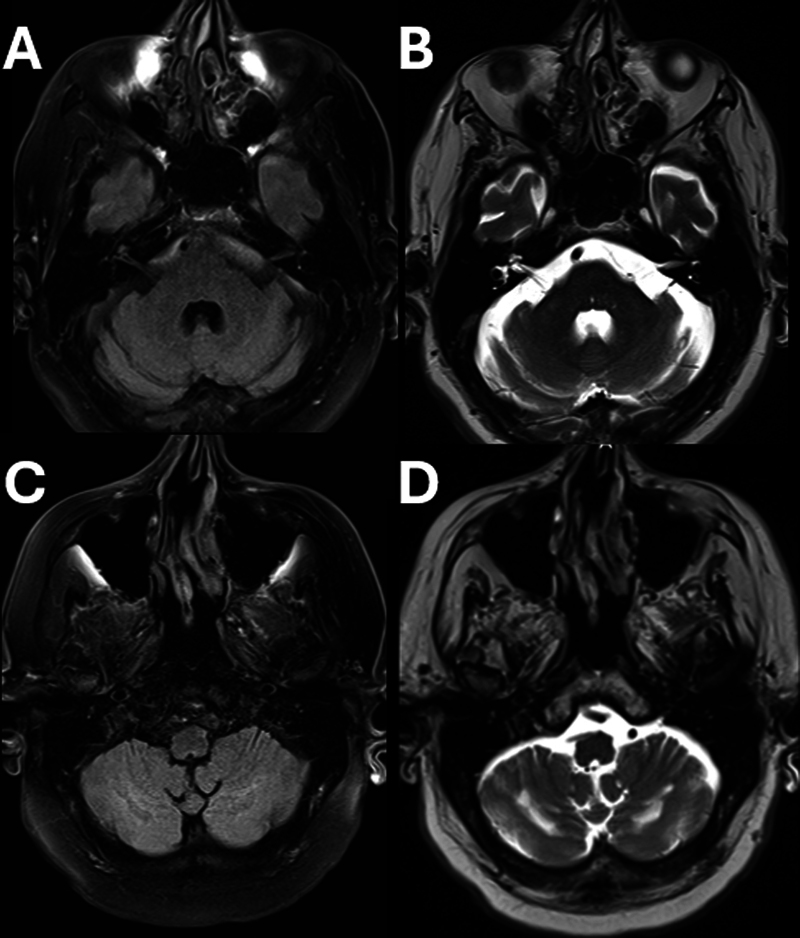
Four-month follow-up brain MRI scan. Axial FLAIR ( **A,C** ) and T2-weighted images ( **B,D** ) demonstrating near-complete resolution of vasogenic edema involving the middle cerebellar peduncles and deep cerebellar white matter, with residual cystic-like changes/small cavitations in the latter.

An 86-year-old man with T-cell large granular lymphocytic leukemia on long-term weekly oral methotrexate presented with progressive imbalance, ataxia, recurrent falls, and altered mental status. Initial neuroimaging ( Figures 1 – 3 ) showed posterior-fossa involvement with abnormalities in the deep cerebellar white matter and middle cerebellar peduncles, consistent with vasogenic edema. Differential diagnoses included atypical posterior reversible encephalopathy syndrome (PRES), 1 John Cunningham (JC) virus-associated progressive multifocal leukoencephalopathy (PML), 2 and metronidazole-induced neurotoxicity, 3 the latter considered less likely due to dentate nuclei sparing. Infectious, metabolic, and neoplastic etiologies were excluded. Methotrexate-induced cerebellar leukoencephalopathy was favored, likely related to folate antagonism and impaired methylation affecting cerebellar white matter. 4 5 6 The neurological symptoms and imaging findings markedly improved after methotrexate discontinuation ( Figure 4 ).
